# CdGAP/ARHGAP31 is regulated by RSK phosphorylation and binding to 14-3-3β adaptor protein

**DOI:** 10.18632/oncotarget.24126

**Published:** 2018-01-10

**Authors:** Ali Ben Djoudi Ouadda, Yi He, Viviane Calabrese, Hidetaka Ishii, Rony Chidiac, Jean-Philippe Gratton, Philippe P. Roux, Nathalie Lamarche-Vane

**Affiliations:** ^1^ Cancer Research Program, Research Institute of the MUHC, Montreal, Quebec, H4A 3J1, Canada; ^2^ McGill University, Department of Anatomy and Cell Biology, Montreal, Quebec, H3A 2B2, Canada; ^3^ Institute for Research in Immunology and Cancer (IRIC), Montreal, Quebec, H3T 1J4, Canada; ^4^ Department of Pharmacology, Faculty of Medicine, Université de Montréal, Department of pharmacology, Montreal, Quebec, H3T 1J4, Canada

**Keywords:** RhoGAPs, RSK, 14-3-3, phosphorylation, cytoskeleton

## Abstract

Cdc42 GTPase-activating protein (CdGAP, also named ARHGAP31) is a negative regulator of the GTPases Rac1 and Cdc42. Associated with the rare developmental disorder Adams-Oliver Syndrome (AOS), CdGAP is critical for embryonic vascular development and VEGF-mediated angiogenesis. Moreover, CdGAP is an essential component in the synergistic interaction between TGFβ and ErbB-2 signaling pathways during breast cancer cell migration and invasion, and is a novel E-cadherin transcriptional co-repressor with Zeb2 in breast cancer. CdGAP is highly phosphorylated on serine and threonine residues in response to growth factors and is a substrate of ERK1/2 and GSK-3. Here, we identified Ser1093 and Ser1163 in the C-terminal region of CdGAP, which are phosphorylated by RSK in response to phorbol ester. These phospho-residues create docking sites for binding to 14-3-3 adaptor proteins. The interaction between CdGAP and 14-3-3 proteins inhibits the GAP activity of CdGAP and sequesters CdGAP into the cytoplasm. Consequently, the nucleocytoplasmic shuttling of CdGAP is inhibited and CdGAP-induced cell rounding is abolished. In addition, 14-3-3β inhibits the ability of CdGAP to repress the E-cadherin promoter and to induce cell migration. Finally, we show that 14-3-3β is unable to regulate the activity and subcellular localization of the AOS-related mutant proteins lacking these phospho-residues. Altogether, we provide a novel mechanism of regulation of CdGAP activity and localization, which impacts directly on a better understanding of the role of CdGAP as a promoter of breast cancer and in the molecular causes of AOS.

## INTRODUCTION

The Rho family of small GTPases holds central functions in cell proliferation, migration, and adhesion [1, 2]. Alterations in *Rho* genes are linked to many human cancers and indicate a role in tumor invasion and metastasis [3–5]. Rho proteins act as molecular switches by cycling between an active GTP- and an inactive GDP-bound state. This GDP/GTP exchange is regulated by guanine nucleotide exchange factors (GEFs), which induce the replacement of GDP by GTP, guanine nucleotide dissociation inhibitors (GDIs) binding and sequestering the GDP-bound form of the GTPase in the cytoplasm, and finally GTPase-activating proteins (GAPs) that stimulate the intrinsic GTPase activity, leading to deactivation of the GTPase [6–8].

Cdc42 GTPase-activating protein (CdGAP, also known as ARHGAP31), a member of the large family of RhoGAPs, negatively regulates the activity of Rac1 and Cdc42, but not RhoA [9, 10]. Recently, the loss of CdGAP in mice unveiled the importance of CdGAP in embryonic vascular development [11]. CdGAP has also been shown to control directional membrane protrusions of migrating osteosarcoma cells [12–14]. In addition, CdGAP mediates transforming growth factor (TGFβ)- and ErbB2-induced cell motility and invasion of breast cancer cells in a GAP-independent manner [15]. Of interest, a quantitative RNA profile analysis of Rho GTPases and their regulators in ErbB2-induced mouse breast tumors revealed Rac1 and CdGAP as the major GTPase and RhoGAP expressed in these tumors, respectively [16]. Recently, we demonstrated that CdGAP acts as a positive modulator of breast tumorigenesis [17]. CdGAP is a large protein, comprising several regulatory domains, each of them being associated with a specific function. Notably, CdGAP consists of an N-terminal GAP domain preceded by a stretch of polybasic residues (PBR) binding to phosphatidylinositol 3,4,5-trisphosphate (PI (3,4,5) P3) that regulates its GAP activity by targeting the protein at the plasma membrane [18]. The N-terminal GAP domain is followed by a basic-rich (BR) central region, a proline-rich domain (PRD) with an extended C-terminal region. The BR region interacts through an atypical basic-rich motif with the SH3D domain of the endocytic scaffolding protein intersectin leading to inhibition of CdGAP activity [19, 20] while the PRD is responsible for the ability of CdGAP to facilitate TGFβ-mediated cell motility and invasion of breast cancer cells [15] and to repress E-cadherin expression [17]. Furthermore, truncating mutations in the terminal exon of the *CdGAP* gene have been identified in patients with a rare developmental disorder, the Adams-Oliver Syndrome (AOS), characterized by the combination of aplasia cutis congenita (ACC) and limb defects [21–23]. These mutations result in the removal of the C-terminal region and part of the PRD of CdGAP, which increase the GAP activity of the truncated proteins through a gain-of-function mechanism [21]. However, the mechanisms underlying the role of the C-terminal region in the control of CdGAP activity remain largely unknown. CdGAP is highly phosphorylated on serine and threonine residues in response to growth factors and is a substrate of extracellular signal-regulated kinase (ERK) and GSK-3. Indeed, phosphorylation of T776 in the PRD by ERK1/2 and GSK-3 negatively regulates the GAP activity of CdGAP [24, 25]. CdGAP was also found to interact with members of the mitogen-activated protein kinase (MAPK) signaling pathway, ERK1/2 and p90 ribosomal protein S6 kinase (RSK) [25]. Mutations of key residues in the ERK docking site impair ERK binding and phosphorylation of CdGAP [25]. Here we report the identification of two important serine residues S1093 and S1163 phosphorylated by RSK, which creates 14-3-3 docking sites in the C-terminal region of CdGAP. We show that 14-3-3β interacts with CdGAP through these phosphoserines and sequesters the protein into the cytoplasm, which inhibits the nucleocytoplasmic shuttling of CdGAP, cell rounding, cell migration, its GAP activity towards Rac1, and its ability to repress E-cadherin expression. In this way, we highlight a novel important mechanism of regulation of CdGAP by 14-3-3 interactions, controlling both GAP-dependent and independent functions of CdGAP in the regulation of cellular morphology and cell migration. Being a critical modulator of breast tumorigenesis [17], targeting 14-3-3-CdGAP interactions offer novel therapeutic perspectives for the treatment of breast cancer. Furthermore, this work provides mechanistic insights into understanding the function of the C-terminal region of CdGAP, lacking in the truncated mutants expressed in AOS patients.

## RESULTS

### CdGAP is a substrate of the AGC family kinases in response to growth factors and mitogens

**Figure 1:**
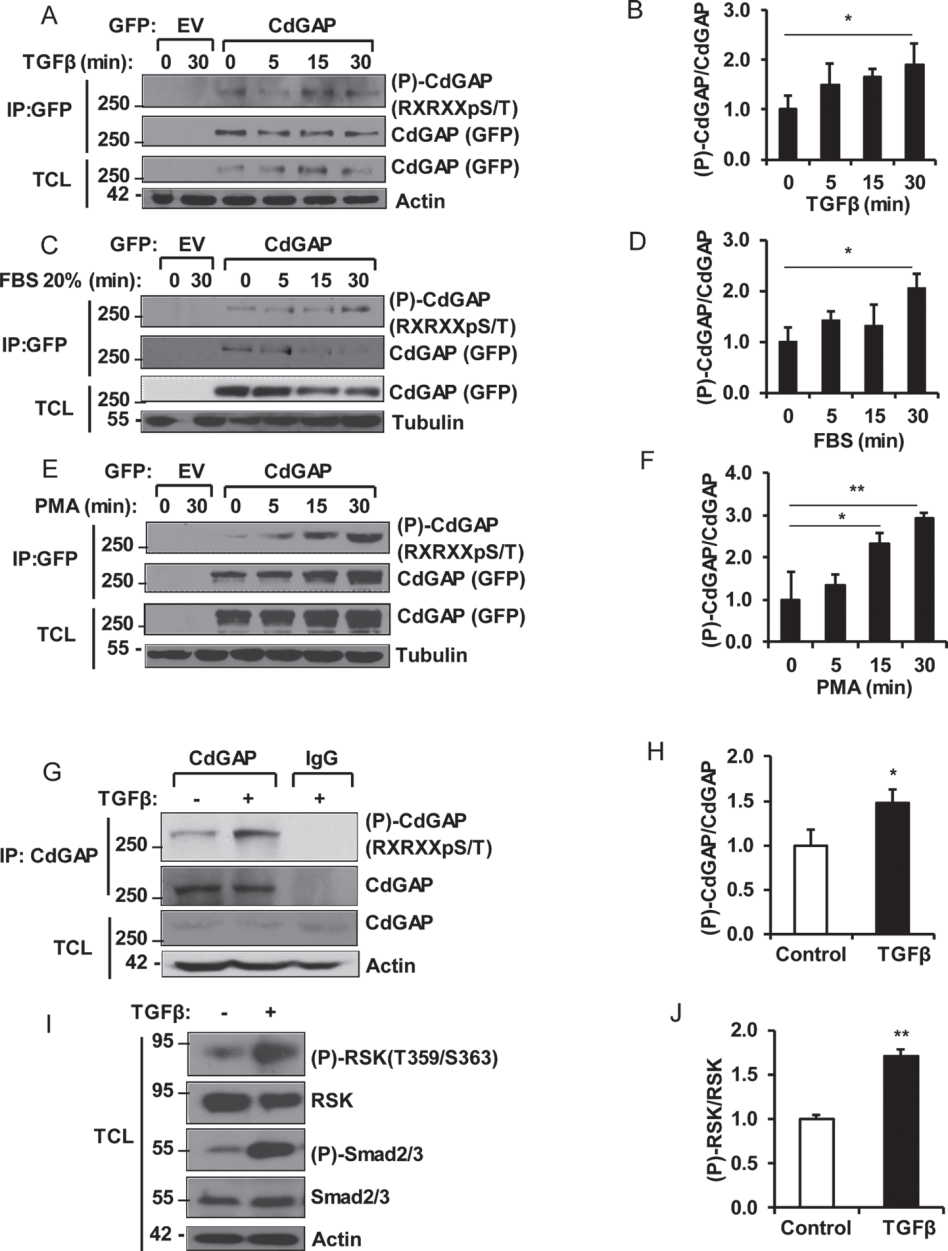
CdGAP is phosphorylated by the AGC family kinases in response to growth factors and mitogens COS-7 cells transfected with empty vector (EV) or GFP-CdGAP were stimulated with TGFβ (5 ng/ml) (**A, B**), FBS 20% (**C, D**), or PMA (200 nM) (**E, F**). GFP-CdGAP proteins were immunoprecipitated (IP) from cell lysates and the phosphorylation of CdGAP (P-CdGAP) on the consensus motif RXRXXpS/T was detected by immunoblotting with the indicated antibodies. TCL, total cell lysates. (B, D, F) Densitometric analysis of P-CdGAP/CdGAP ratio is represented as the fold change relative to 0 min of stimulation from A, C, and E. (^*^p < 0.05, ^**^p < 0.01, unpaired Student’s *t* test). (**G**) Endogenous CdGAP was IP from lysates of mouse mammary epithelial (NMuMG) cells, stimulated with TGFβ (5 ng/ml) for 30 min with anti-CdGAP antibodies or rabbit IgG as control. IP proteins and total cell lysates (TCL) were resolved by SDS-PAGE and immunoblotted with the indicated antibodies. (**H**) Densitometric analysis of P-CdGAP/CdGAP ratio from G. (^*^p < 0.05, unpaired Student’s *t* test). (**I**) Total cell lysates (TCL) from NMuMG cells stimulated with TGFβ (5ng/ml) for 30min were resolved by SDS-PAGE and immunoblotted with the indicated antibodies. (**J**) Densitometric analysis of (P)-RSK/RSK from I. (^**^p < 0.01, unpaired student’s *t* test).

To determine if AGC family kinases, such as Akt and RSK [26, 27], phosphorylate CdGAP in response to agonists of the Ras/MAPK pathway, we used the phosphorylation site-specific antibody recognizing the consensus motif Arg/Lys-X-Arg/Lys-X-X-pSer/Thr (RXRXXpS/T) found in substrates of the AGC family kinases [26, 27]. COS-7 fibroblast cells transfected with GFP-tagged CdGAP were serum-starved overnight before stimulation with TGFβ, serum, or phorbol ester (phorbol-12-myristate-13-acetate, PMA) for 5 to 30 minutes before harvesting. Immunoprecipitated CdGAP was then examined for phosphorylation by immunoblotting using the anti-RXRXXpS/T antibodies. CdGAP phosphorylation at the basic consensus motif was increased in response to the agonists and peaked at 30 minutes after stimulation (Figure 1A–1F).

We next investigated whether endogenous CdGAP is phosphorylated by AGC kinases in response to TGFβ stimulation of mammary epithelial cells. We found that CdGAP phosphorylation at the basic consensus motif was significantly increased after 30 minutes of TGFβ stimulation (Figure 1G and 1H). CdGAP phosphorylation was concomitant with the phosphorylation of RSK (T359/S363) and Smad2/3 (Figure 1I and 1J), suggesting that activation of RSK by the canonical TGFβ pathway may be responsible of CdGAP phosphorylation on basic consensus motifs. Altogether, these data demonstrate that CdGAP is a substrate of the AGC family kinases, which likely implicate RSK activation in response to growth factor and mitogen stimulation.

### Identification of S1093 and S1163 as RSK-dependent phosphorylation sites

**Figure 2:**
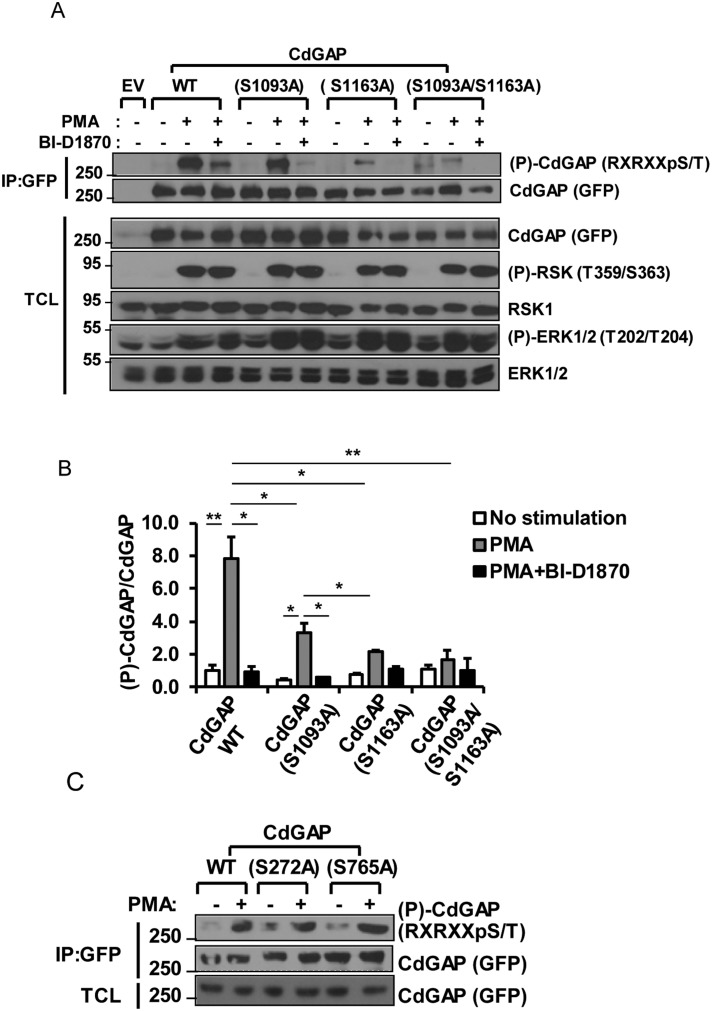
S1093 and S1163 are RSK-dependent phosphorylation sites (**A**) COS-7 cells transfected with empty vector (EV), wild-type (WT) CdGAP or CdGAP point mutants with RSK1 constructs were treated with PMA in the presence or absence of the RSK inhibitor BI-D1870 as indicated. GFP-CdGAP proteins were IP from total cell lysates (TCL). IP proteins and TCL were resolved by SDS-PAGE and immunoblotted with the indicated antibodies. (**B**) Densitometric analysis of (P)-CdGAP/CdGAP from A. (^*^p < 0.05, ^**^p < 0.01, unpaired student’s *t* test). (**C**) COS-7 cells were transfected with the indicated CdGAP constructs and analyzed as in A.

Because we have previously shown that RSK interacts and directly phosphorylates CdGAP *in vitro* [25], we next sought to determine whether RSK phosphorylates CdGAP at the basic consensus sites. COS-7 cells co-transfected with GFP-CdGAP wild-type (WT) and RSK1 were treated with the RSK inhibitor (BI-D1870) that selectively blocks RSK activity [28]. Treatment of cells with BI-D1870 prior to PMA stimulation significantly reduced CdGAP phosphorylation at the basic consensus site (Figure 2A and 2B), showing that RSK induces CdGAP phosphorylation at basic consensus sites in response to PMA.

To identify CdGAP residues phosphorylated by RSK, we analyzed the basic consensus residues surrounding all Ser/Thr amino acids in the CdGAP protein sequence, with at least one Arg residue in the −3 position. According to the RSK consensus phosphorylation sequence determined using an oriented peptide library [29], we identified 8 potential phosphorylation sites (Table 1). Based on the number of phosphoproteomic studies reporting these potential CdGAP phosphorylation sites (http://www.phosphosite.org/), we chose to replace each of the 4 residues Ser272, Ser765, Ser1093, and Ser1163 to an alanine residue. We found that the mutation of Ser272 and Ser765 located in the BR and PRD, respectively, did not significantly affect CdGAP phosphorylation compared to wild type CdGAP in response to PMA treatment of COS-7 cells using anti-RXRXXpSer/Thr antibodies (Figure 2C). However, the phosphorylation of CdGAP-S1093A and –S1163A was significantly reduced compared to wild-type CdGAP in response to PMA treatment whereas the phosphorylation of the double mutant CdGAP-S1093A/S1163A was almost completely absent (Figure 2A and 2B). Treatment of the cells with the RSK inhibitor BI-D1870 prior to PMA completely inhibited the phosphorylation of the single mutants and the double mutant CdGAP-S1093A/S1163A (Figure 2A and 2B). Collectively, these data demonstrate that Ser1093 and Ser1163 located in the C-terminal region of CdGAP are major phosphorylation sites targeted by RSK in response to PMA stimulation.

**Table 1:** Potential RSK phosphorylation sites identified in the CdGAP protein sequence

Ranking No. ^a^	Residue *Mouse (Human)*	CdGAP sequence *Mouse (Human)*	CdGAP-domain	Records^b^
1	Ser1163^c^ (Ser1178)	TLTGRRNpSAPVSVSA (ALTGRRNpSAPVSVSA)	CT	25
2	Ser312 (Ser315)	FNLGRSGpSDSKSKLS (FNLGRSGpSDSKSKLS)	BR	0
3	Ser1093^c^ (Ser1106)	KGKHRPSpSLNLDSAT (KGKNRPSpSLNLDPAI)	CT	3
4	Ser323 (Ser327)	SKLSRNGpSVFVRGQR (SKLSRNGpSVFVRGQR)	BR	2
5	Ser765 (Ser778)	IGGPRNLpSPPLTPAP (VGGPGNLpSPPLPPAP)	PRD	14
6	Ser1331 (Ser1346)	SRPGRPQpSLILFPIM (SRPGRPQpSLILFSPP)	CT	2
7	Ser272 (Ser272)	RKERRENpSLPEIVPP (RKERRENpSLPEIVPP)	BR	18
8	Ser998 (Ser1011)	LKAFREFpSGLKGLEV (LRSFREFpSGLKGAEA)	CT	0

^a^8 potential phosphorylation sites were identified according to the RSK consensus phosphorylation sequence determined using an oriented peptide library (Galan *et al*., 2014) (ranked from the highest to the lowest).

^b^Number of phosphoproteomic studies reporting the phosphorylated residue according to phosphosite.org.

^c^Phosphorylated in response to VEGF and /or Angiopoietin-1 (Chidiac *et al*., 2016).

BR, basic region; PRD, proline- rich region. CT, C-Terminal region.

### 14-3-3 adaptor proteins isoforms β and σ interact with CdGAP

**Figure 3:**
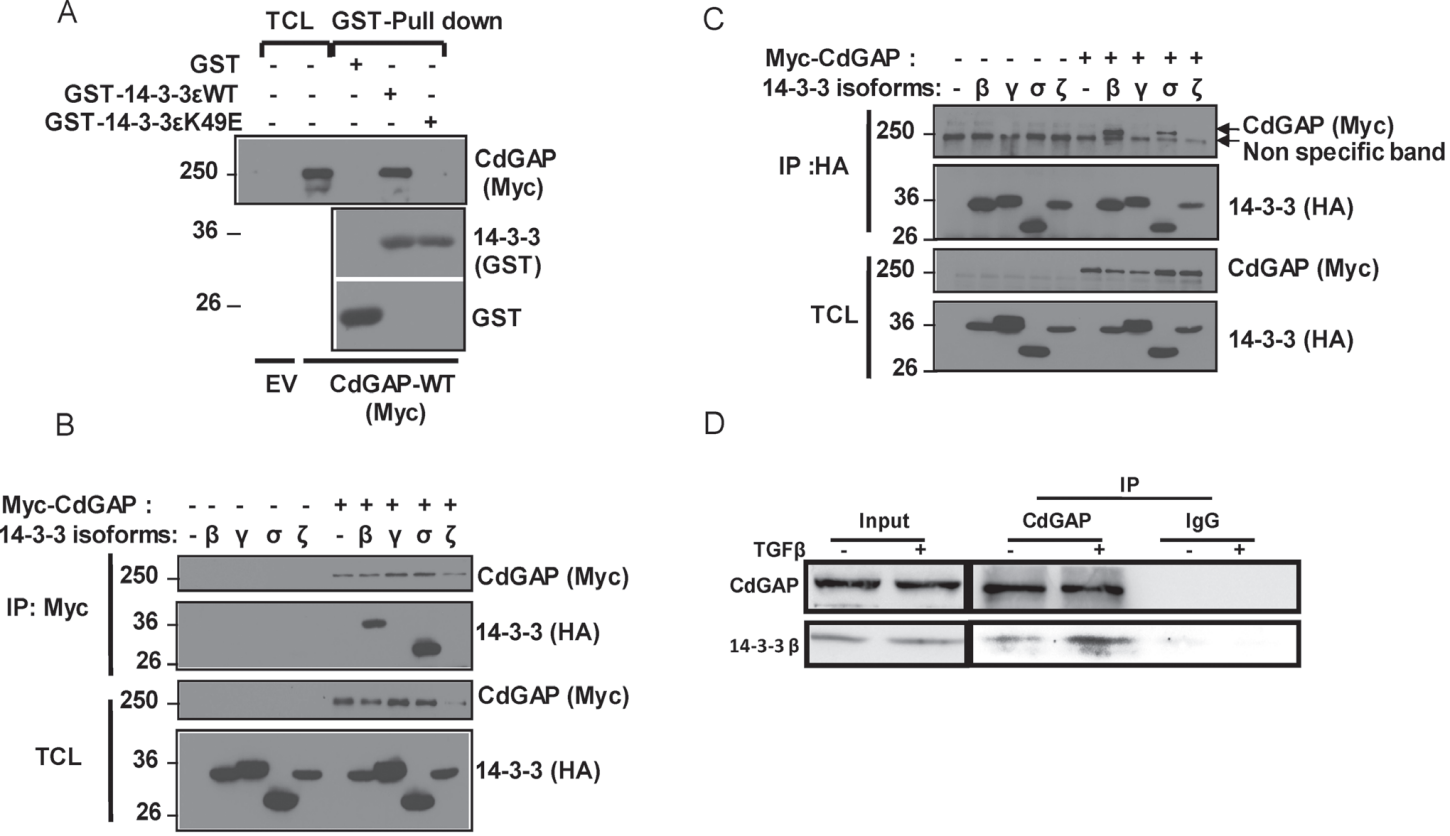
14-3-3 adaptor proteins isoforms β and σ interact with CdGAP (**A**) HEK293 cells were transfected with empty vector (EV) or myc-CdGAP constructs. Proteins from total cell lysates (TCL) were pulled down with GST, GST-14-3-3e wild type (WT) or mutant 14-3-3eK49E proteins. Associated proteins and TCL were resolved by SDS-PAGE and analyzed by immunoblotting using the indicated antibodies. (**B** and **C**) HEK293 cells were transfected with myc-CdGAP and HA-14-3-3 isoform constructs as indicated. Myc-CdGAP proteins (B) or HA-14-3-3 (C) proteins were IP from total cell lysates (TCL), resolved by SDS-PAGE and immunoblotted using anti-Myc and anti-HA antibodies. (**D**) CdGAP was IP from lysates of MDA-MB-231 cells stimulated with TGFβ (5 ng/ml) with anti-CdGAP antibodies or rabbit IgGs as a control. IP proteins and total cell lysates (input) were immunoblotted with the indicated antibodies.

**Figure 4:**
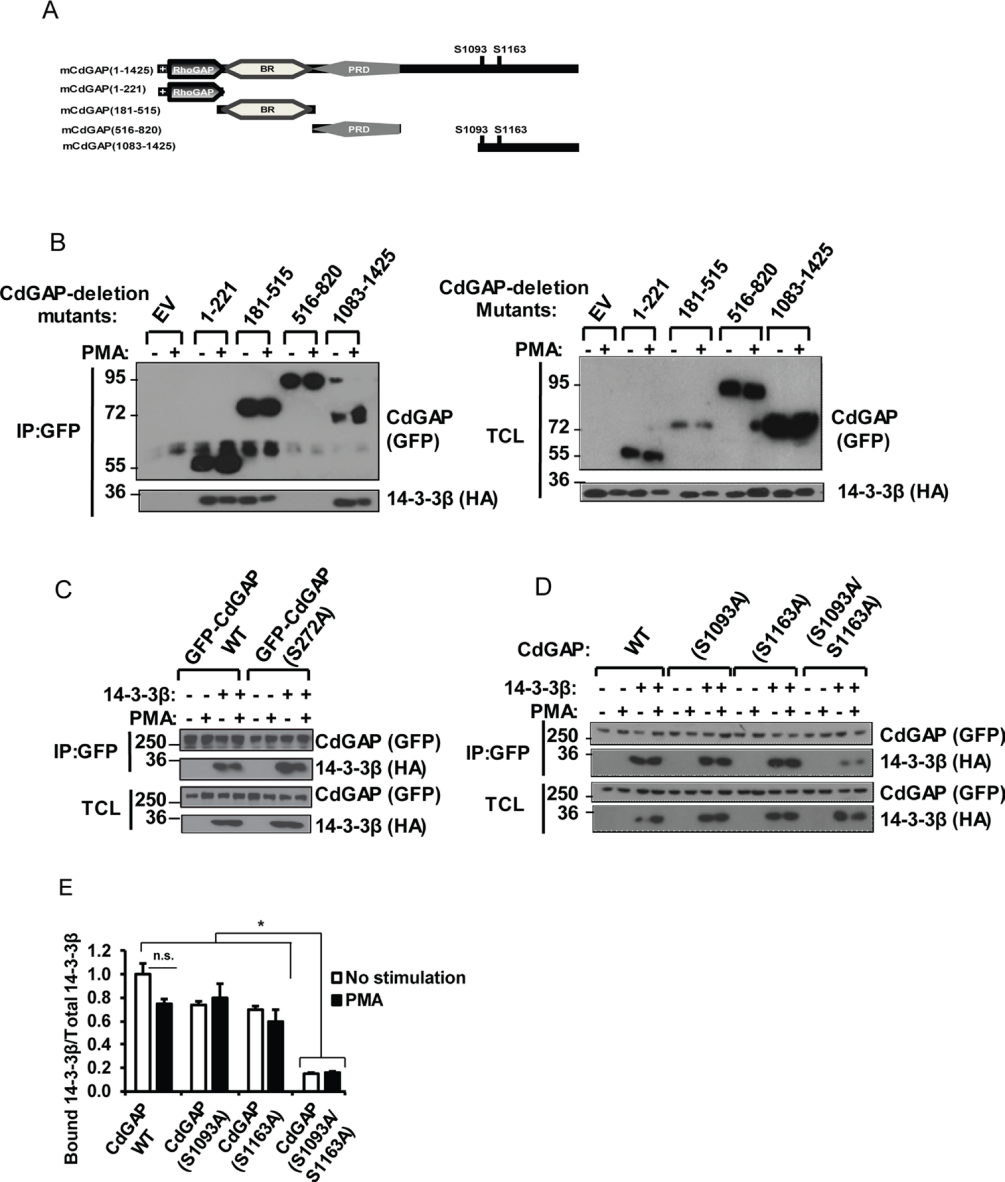
The RSK-dependent phosphorylation residues S1093 and S1163 are required for the interaction between CdGAP and 14-3-3β (**A**) Schematic representation of mouse CdGAP (mCdGAP) deletion mutants, including the major RSK target residues. +, stretch of polybasic residues; rhoGAP, GTPase-activating protein; BR, basic region; PRD, proline-rich domain. (**B**) COS-7 cells were transfected with empty vector (EV) or GFP-CdGAP deletion mutant constructs with HA-14-3-3β and treated with PMA as indicated. GFP-CdGAP proteins were immunoprecipitated (IP) from total cell lysates (TCL). IP proteins and TCL were resolved by SDS-PAGE and immunoblotted with the indicated antibodies. (**C** and **D**) COS-7 cells were transfected with the indicated CdGAP constructs with HA-14-3-3β and analyzed as in B. (**E**) Densitometric analysis of bound 14-3-3β to CdGAP/total 14-3-3β from D. (^*^p < 0.05, n.s., not significant, unpaired student’s *t* test).

Because the minimum RSK consensus motif RXXpSer/Thr overlaps with the 14-3-3 mode 1 binding site RXXpSer/ThrXP [30–32], we next investigated whether CdGAP interacts with 14-3-3 proteins. Myc-tagged CdGAP was expressed in HEK293 cells and subjected to a GST-14-3-3 pull-down. Consistent with a phospho-dependent interaction, CdGAP interacted with wild-type 14-3-3ε but not with the mutant 14-3-3εK49E, which shows reduced binding to phosphorylated substrates [31] (Figure 3A). Then, we examined which 14-3-3 isoforms interact with CdGAP in co-immunoprecipitation assays. Myc-tagged CdGAP was immunoprecipitated from HEK293 cells expressing Myc-CdGAP together with the HA-tagged 14-3-3 isoforms. CdGAP interacted specifically with 14-3-3β and σ isoforms (Figure 3B). Similarly, immunoprecipitated HA-tagged 14-3-3 isoforms showed an interaction between 14-3-3β or σ with CdGAP (Figure 3C). Additionally, we detected an interaction between the endogenous CdGAP and 14-3-3β proteins, which was increased in response to TGFβ stimulation in MDA-MB-231 human breast cancer cells (Figure 3D). To delineate the regions of CdGAP permitting the association between CdGAP and 14-3-3, GFP-CdGAP deletion mutants (Figure 4A) were expressed in COS-7 cells and the interaction with 14-3-3β was assessed by immunoprecipitation in unstimulated or PMA-treated cells (Figure 4B). In this assay, the N-terminal PBR-GAP fragment (1-221), the BR (181-515) and C-terminal domains (1083-1425) but not the PRD (516-820) interacted with 14-3-3β (Figure 4B). However, we did not observe an increased interaction in PMA-stimulated cells (Figure 4B). We next examined the interaction between 14-3-3β and CdGAP phospho-mutants co-expressed in COS-7 cells unstimulated or treated with PMA. CdGAP-S272A was still able to interact with 14-3-3β (Figure 4C), suggesting that this basic consensus motif was not responsible for the binding of CdGAP-BR to 14-3-3β. Conversely, the interaction between CdGAP double mutant S1093A/S1163A and 14-3-3β was greatly reduced compared to wild-type CdGAP or to each single CdGAP mutant in unstimulated and PMA-treated cells (Figure 4D and 4E). Therefore, these results show that Ser1093 and Ser1163 residues in the C-terminal region of CdGAP create 14-3-3 binding sites.

### 14-3-3β regulates CdGAP subcellular localization and inhibits CdGAP-mediated cell rounding

**Figure 5:**
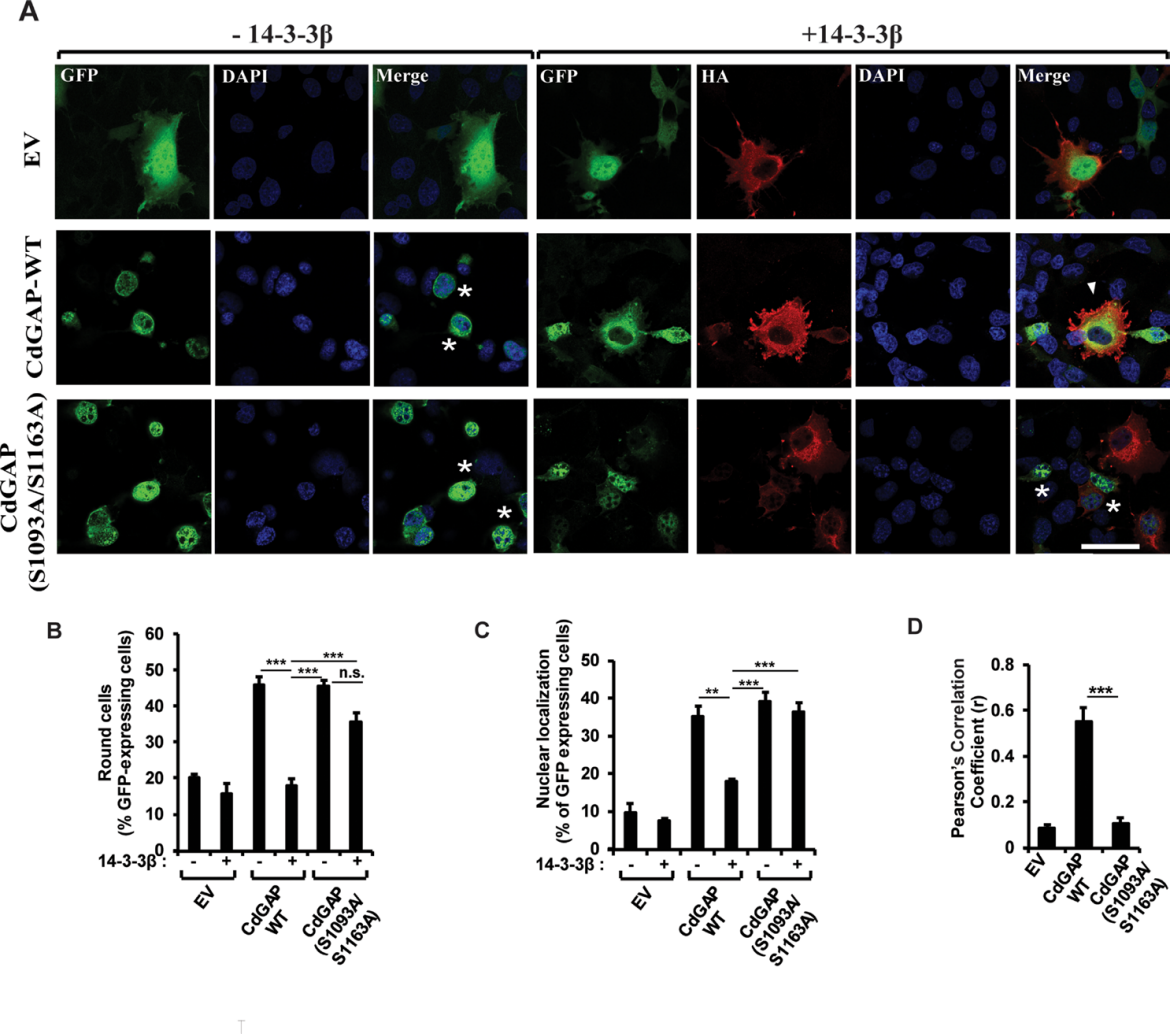
14-3-3β regulates CdGAP subcellular localization and inhibits CdGAP-mediated cell rounding (**A**) GFP-CdGAP constructs with or without 14-3-3β were transfected into COS-7 cells before fixation. CdGAP and 14-3-3β localizations were assessed by indirect immunofluorescence and confocal microscopy. CdGAP (green), 14-3-3β (red), nucleus (blue) were visualized using anti-GFP, anti-HA antibodies, and DAPI staining, respectively. Bar, 50μm. star, round cells, arrowheads, cytoplasmic localization of CdGAP. (**B** and **C**) The percentage of GFP-expressing cells showing cell rounding (B) or CdGAP nuclear localization (C) was calculated manually using Metamorph software. Cells with nuclei occupying ≥ 50% of the total cell area were counted as being round and cells with ≥70% of their GFP staining in the nucleus were counted as nuclear localization. More than 100 GFP-positive cells were counted in at least three independent experiments. (^**^p < 0.01, ^***^p < 0.001, n.s., non-significant, unpaired student’s *t* test). (**D**) The correlation between CdGAP and 14-3-3β fluorescence intensities in A was measured with ZEN2010 software using Pearson’s correlation coefficient (r). At least 30 cells from three independent experiments were analyzed (^***^p < 0.001, unpaired student’s *t* test).

We have previously demonstrated that the expression of CdGAP in various cell types induces cell rounding in a GAP-dependent manner [10, 14, 18, 21]. Thus, we assessed whether 14-3-3β regulates the ability of CdGAP to induce cell rounding and its subcellular localization by microscopy. Wild-type CdGAP was expressed alone or with 14-3-3β into fibroblast cells and the percentage of cells showing a rounded phenotype was determined (Figure 5). As previously reported, CdGAP-WT induced cell rounding in 45% of transfected cells compared to 20% of control GFP-transfected cells (Figure 5A and 5B). We confirmed by FACS analysis that the cell rounding effect induced by CdGAP was not caused by a change in the cell cycle (Supplementary Figure 1). Co-expression of 14-3-3β and CdGAP-WT abolished the ability of CdGAP to induce cell rounding, showing a flat and elongated phenotype (Figure 5A and 5B). In addition, we determined that CdGAP showed a nuclear localization in 35% of transfected cells (Figure 5A and 5C) whereas the expression of 14-3-3β with CdGAP significantly reduced the nuclear localization of CdGAP to 15% of transfected cells (Figure 5A and 5C). We assessed the degree of co-association of 14-3-3β and CdGAP in the cytoplasm by calculation of the mean Pearson’s correlation coefficient between 14-3-3β and CdGAP. By this mean, we observed a significant colocalization of CdGAP with 14-3-3β in the cytoplasm (r = 0.55+/−0.06) (Figure 5D). We next examine the effect of 14-3-3β on the localization of the double mutant CdGAP-S1093A/S1163A and its ability to induce cell rounding. Similar to wild-type CdGAP, the mutant CdGAP-S1093A/S1163A induced cell rounding in 45% of transfected cells (Figure 5A and 5B). However, the expression of 14-3-3β with the double mutant did not affect its ability to induce cell rounding (Figure 5A and 5B). Furthermore, 14-3-3β did not affect the percentage of transfected cells with CdGAP-S1093A/S1163A nuclear localization (Figure 5A and 5C) and consequently, 14-3-3β did not colocalize with CdGAP-S1093A/S1163A (r = 0.10+/−0.02) (Figure 5D). Together, these data show that 14-3-3β sequesters CdGAP into the cytoplasm and inhibits its ability to induce cell rounding, which is dependent on the RSK-mediated phosphorylation sites Ser1093 and Ser1163.

### 14-3-3β docking sites negatively regulate the GAP activity of CdGAP towards Rac1

**Figure 6:**
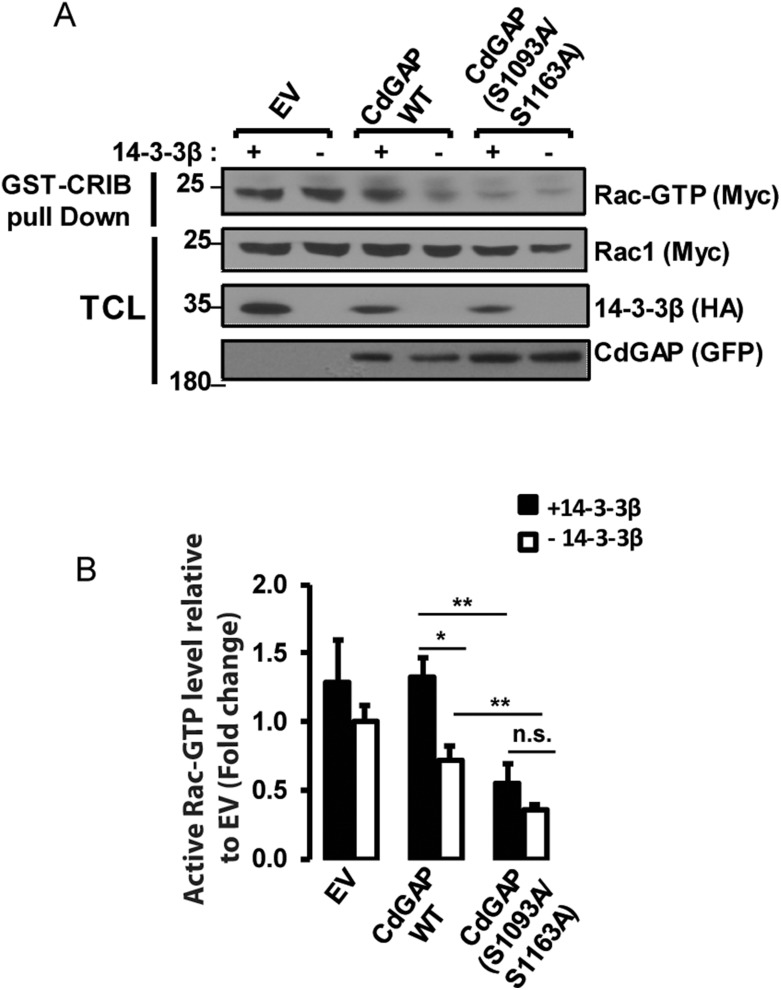
14-3-3β negatively regulates the GAP activity of CdGAP towards Rac1 (**A**) HEK293 cells were transfected with pRK5-mycRac1 with empty vector (EV), the indicated GFP-CdGAP constructs, and HA-14-3-3β as indicated. GTP-loaded Rac1 was pulled down from total protein lysates (TCL) by GST-CRIB. GTP-bound Rac1, total Rac1, and the indicated proteins were detected by immunoblotting. (**B**) Densitometric ratio of GTP-bound Rac1/total Rac1 relative to EV from A. (^*^p < 0.05, ^**^p < 0.01, n.s., non-significant, unpaired student’s *t* test).

We next sought to determine whether 14-3-3β directly modulates CdGAP activity towards Rac1. To this end, we performed pull-down assays with the Cdc42/Rac1 interactive binding (CRIB) domain of PAK fused to GST to assess the levels of active GTP-Rac1 in HEK293 cell extracts. As expected, overexpression of CdGAP led to a significant reduction in GTP-Rac1 levels (*p* < 0.05), whereas the expression of 14-3-3β with CdGAP resulted in the inhibition of CdGAP activity with higher levels of GTP-Rac1 *(P* < 0.05) comparable to controls transfected with empty vector and 14-3-3β (Figure 6A and 6B). To examine whether the 14-3-3β docking sites in the C-terminal region of CdGAP regulate CdGAP activity, we determined the GAP activity of the double mutant CdGAP-S1093A/S1163A alone or with 14-3-3β. The expression of CdGAP-S1093A/S1163A resulted in a significant higher GAP activity with lower levels of GTP-Rac1 (*P* < 0.01), whereas co-expression with 14-3-3β did not significantly modulate its GAP activity (Figure 6A and 6B). Together with the previous findings that 14-3-3β abolished CdGAP-mediated cell rounding (Figure 5A and 5B), these results support the hypothesis that 14-3-3β inhibits the GAP activity of CdGAP towards Rac1 through interaction with the RSK-dependent phosphorylation sites Ser1093 and Ser1163.

### The AOS-related CdGAP mutant proteins show a reduced interaction with 14-3-3β

**Figure 7:**
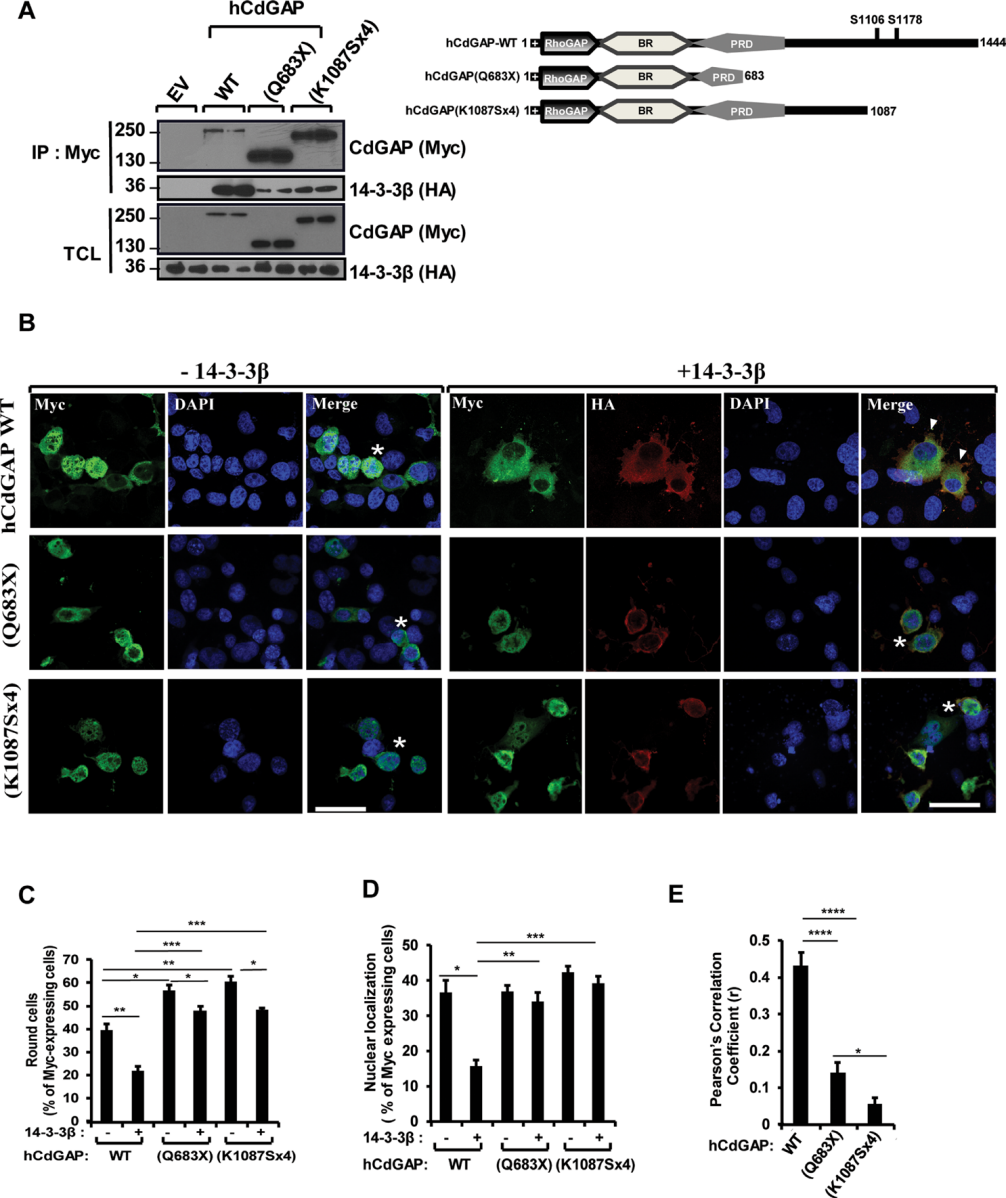
Reduced interaction and modulation of AOS-related CdGAP mutant proteins by 14-3-3β (**A**) COS-7 cells were transfected with HA-14-3-3β together with empty vector (EV) or myc-human CdGAP (hCdGAP) constructs as indicated. Myc-hCdGAP proteins were IP from total cell lysates (TCL) in duplicates, resolved by SDS-PAGE and immunoblotted using anti-Myc and anti-HA antibodies. Schematic representation of hCdGAP-WT and AOS-associated mutants. (**B**) myc-hCdGAP constructs with or without 14-3-3β were transfected into COS-7 cells before fixation. CdGAP and 14-3-3β localizations were assessed by indirect immunofluorescence and confocal microscopy. hCdGAP (green),14-3-3β (red), nucleus (blue) were visualized using anti-GFP, anti-HA antibodies, and DAPI staining, respectively. Bar, 50μm. star, round cells, arrowheads, cytoplasmic localization of CdGAP. (C and D) The percentage of GFP-expressing cells showing cell rounding (**C**) or hCdGAP nuclear localization (**D**) was calculated manually using Metamorph software as in Figure 5 (^*^p < 0.05, ^**^p < 0.01, ^***^p < 0.001, unpaired student’s *t* test). (**E**) The correlation between hCdGAP and 14-3-3β fluorescence intensities in B was measured with ZEN2010 software using Pearson’s correlation coefficient (r). At least 30 cells from three independent experiments were analyzed (^*^p < 0.05, ^****^p < 0.0001, unpaired student’s *t* test).

Because the AOS-related CdGAP-Q683X and –K1087Sx4 mutants are lacking the C-terminal region and show an increased GAP activity [21], we next determined the interaction between 14-3-3β and the AOS-related CdGAP mutant proteins in co-immunoprecipitation assays (Figure 7A). Myc-tagged human CdGAP (hCdGAP) wild-type, CdGAP-Q683X or –K1087Sx4 were immunoprecipitated from COS-7 cells expressing HA-tagged 14-3-3β. Both AOS-related CdGAP mutants showed a reduced, but not completely abolished, interaction with 14-3-3β compared to the wild-type hCdGAP (Figure 7A). Consistent with the previous findings that 14-3-3β can also interact with the N-terminal GAP and BR of mouse CdGAP (Figure 4B), these data suggest that 14-3-3β association with human CdGAP may be in part mediated by the N-terminus and BR, though the C-terminal region of human CdGAP lacking in the AOS-related mutants is required to mediate an efficient interaction with 14-3-3β.

### 14-3-3β is unable to modulate AOS-related CdGAP mutant localization and activity

We next explored the role of 14-3-3β on the subcellular localization and the ability of the AOS-related mutants to induce cell rounding. As previously reported [21], human CdGAP induced cell rounding in 40% of transfected cells, whereas the AOS-related mutants showed a significant increased rounded phenotype with 55% and 60% of transfected cells for CdGAP-Q683X and –K1087Sx4 expression, respectively (Figure 7B and 7C). In addition, human CdGAP-WT and AOS-related mutants showed a similar nuclear localization in 38% to 42% of transfected cells (Figure 7B and 7D). Co-expression of 14-3-3β inhibited the ability of hCdGAP-WT to induce cell rounding, whereas it has a small but significant capability of reducing the ability of AOS-related mutant proteins to induce cell rounding (Figure 7B and 7C). Furthermore, 14-3-3β decreased the percentage of cells with nuclear hCdGAP-WT localization to 15%, whereas it has no effect on the localization of the AOS-related mutants (Figure 7B and 7D). Consequently, we did not find a significant co-localization between the AOS-related mutants and 14-3-3β by assessing the degree of co-association with the Pearson’s correlation coefficient, whereas hCdGAP-WT co-localized with 14-3-3β with an r = 0.43+/−0.03 (Figure 7E). Collectively, these results demonstrate that the negative regulation of CdGAP activity by 14-3-3β is impaired for the AOS-related protein mutants, which correlates with its increased GAP activity [21] and ability to induce cell rounding.

### 14-3-3β inhibits the ability of CdGAP to repress the E-cadherin promoter and to induce cell migration

**Figure 8:**
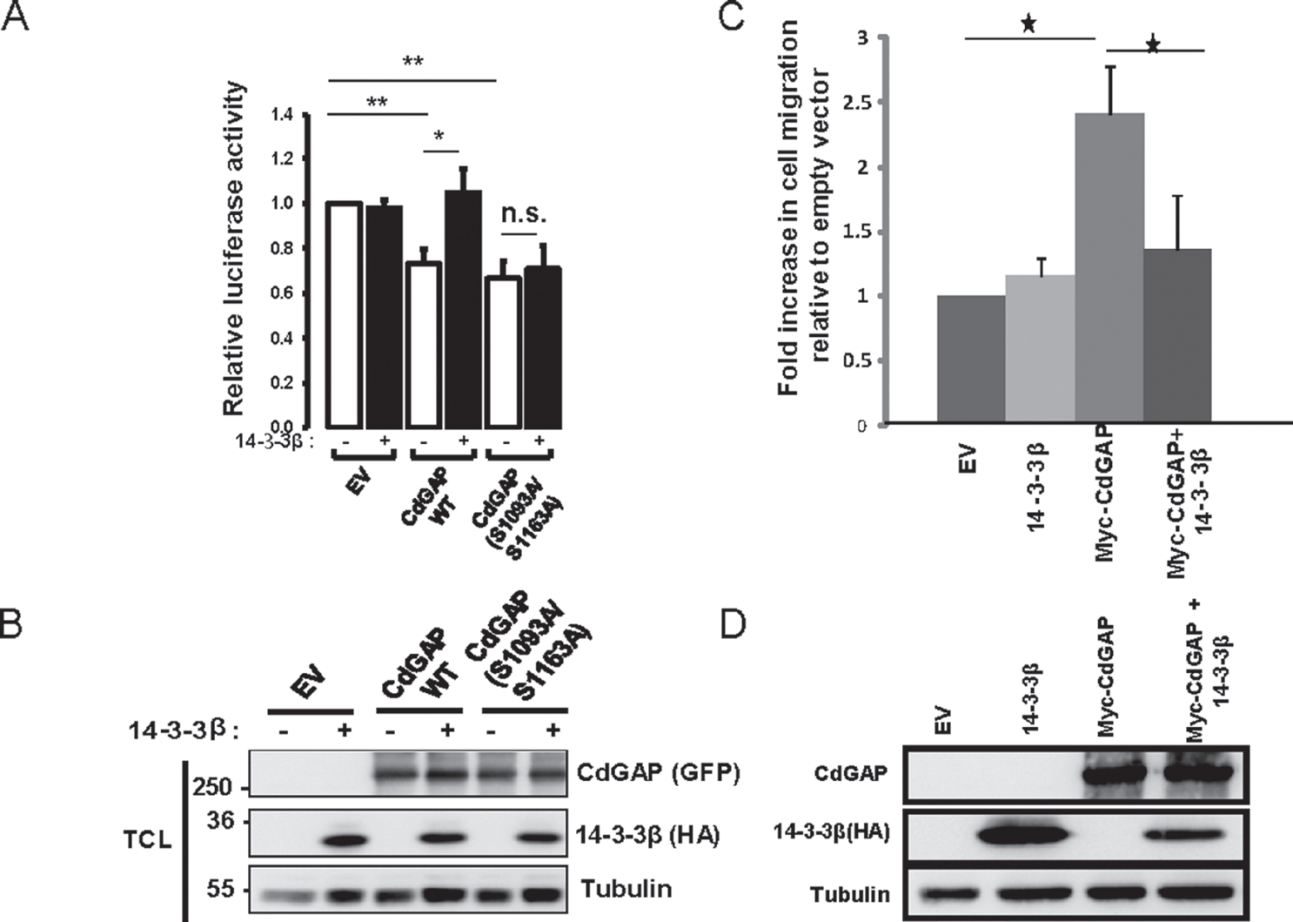
14-3-3β blocks the ability of CdGAP to repress the E-cadherin promoter and to induce cell migration (**A**) E-cadherin promoter luciferase assays were performed in HEK293 cells transfected with empty vector (EV), HA-14-3-3β, wild-type (WT) CdGAP or CdGAP point mutants as indicated. Values are relative to that of HEK293 cells transfected with EV (^*^p < 0.05, ^**^p < 0.001, n.s., not significant, unpaired student’s *t* test). (**B**) Total cell lysates (TCL) from A resolved by SDS-PAGE and immunoblotted with the indicated antibodies. (**C**) Migration assays were performed in HEK293 cells transfected with empty vector (EV), HA-14-3-3β or Myc-CdGAP as indicated. (^*^p < 0.05, unpaired student’s *t* test). (**D**) Total cell lysates from C resolved by SDS-PAGE and immunoblotted with the indicated antibodies.

**Figure 9:**
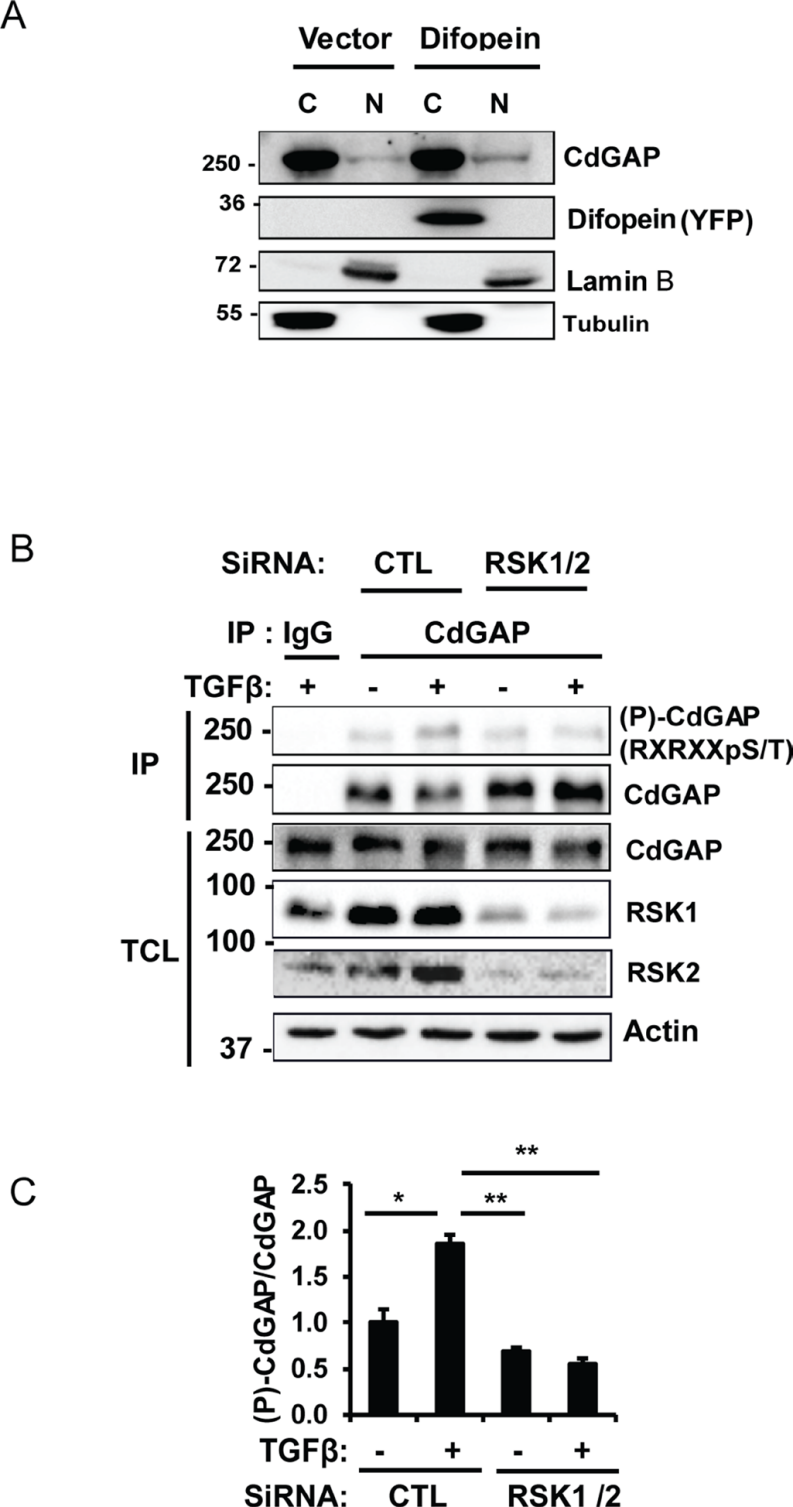
CdGAP is phosphorylated on basic consensus sites in an RSK1/2-dependent manner in breast cancer cells (**A**) Nuclear (N) and cytoplasmic (C) fractions were isolated from MDA-MB-231 cells transfected with empty vector (Vector) or with the 14-3-3 antagonist YPF-Difopein. Each fraction was immunoblotted with the indicated antibodies. Tubulin and Lamin B1 were used as specific markers of the cytoplasmic and nuclear fractions, respectively. (**B**) Endogenous CdGAP was immunoprecipitated (IP) from total cell lysates (TCL) of MDA-MB-231 cells transfected with control (CTL) siRNA or siRNAs targeting RSK1/2 and treated with TGFβ (5 ng/ml) for 30 min. IP proteins and TCL were resolved by SDS-PAGE and immunoblotted with the indicated antibodies. (**C**) Densitometric analysis of (P)-CdGAP/CdGAP from B. (^*^p < 0.05, ^**^p < 0.01, unpaired student’s *t* test).

We have recently uncovered a previously unknown nuclear function for CdGAP where it cooperates in a GAP-independent manner with the transcriptional repressor Zeb2 to regulate the repression of E-cadherin expression in breast cancer cells [17]. We next sought to determine whether 14-3-3β regulates the transcriptional activity of CdGAP by employing a luciferase reporter construct controlled by the E-cadherin promoter [17]. As previously reported [17], overexpression of CdGAP was able to repress the E-cadherin promoter activity, whereas co-expression with 14-3-3β completely inhibited the ability of CdGAP to repress the E-cadherin promoter activity (Figure 8A and B). Similarly, the expression of CdGAP in HEK293 cells induced a 2.5-fold increase in cell migration, which was completely abrogated in the presence of 14-3-3β overexpression (Figure 8C and 8D). Furthermore, the CdGAP-S1093A/S1163A mutant was also able to repress the E-cadherin promoter activity, but it was not regulated by the expression of 14-3-3β (Figure 8A and 8B). Therefore, these results show that 14-3-3β regulates the transcriptional activity of CdGAP through the regulation of its subcellular localization; consistent with the above findings that 14-3-3β sequesters CdGAP in the cytoplasm through interaction with the RSK-dependent phosphorylation sites Ser1093 and Ser1163 (Figure 5). We next assessed by subcellular fractionation experiments if 14-3-3 proteins regulate the subcellular localization of endogenous CdGAP in MDA-MB-231 breast cancer cells, which express high levels of CdGAP and low levels of E-cadherin [17] (Figure 9A). Endogenous CdGAP was mostly found in the cytoplasmic fraction of MDA-MB-231 cell lysates as reported earlier [17], whereas the expression of the potent 14-3-3 antagonist difopein [29, 33] led to an increase of endogenous CdGAP into the nuclear fraction (Figure 9A). Furthermore, to determine whether CdGAP is phosphorylated at the basic consensus motif by RSK1/2 in MDA-MB-231 cells, we transfected synthetic siRNAs targeting human RSK1 and RSK2 [34] in MDA-MB-231 cells (Figure 9B). We found that TGFβ stimulation induced a two-fold increase in the phosphorylation of endogenous CdGAP at the basic consensus motif in MDA-MB-231 cells transfected with control siRNAs. However, TGFβ-induced CdGAP phosphorylation was abolished in RSK1/2-depleted cells (Figure 9B and 9C). Altogether, these results show that endogenous CdGAP is phosphorylated on basic consensus sites in an RSK1/2-dependent manner, which supports the regulation of the nucleocytoplasmic shuttling of CdGAP by the adaptor 14-3-3 proteins in MDA-MB-231 breast cancer cells.

## DISCUSSION

**Figure 10:**
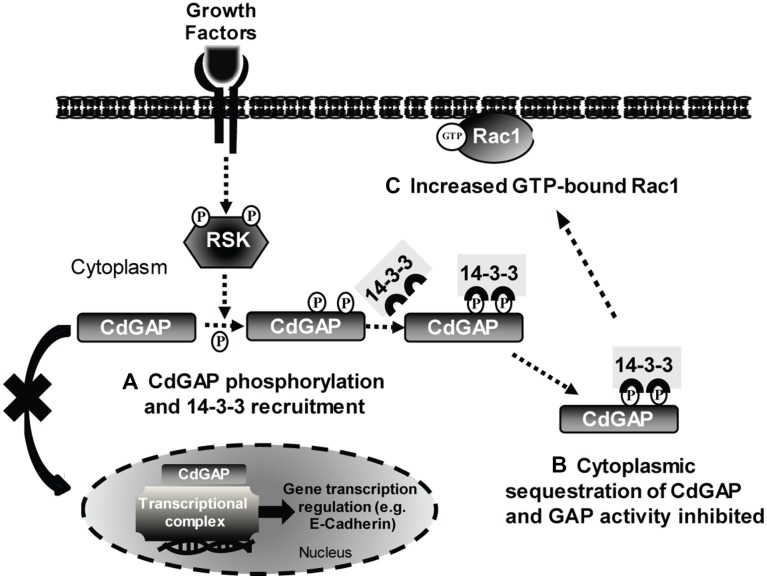
Model of CdGAP regulation by 14-3-3 adaptor proteins (**A**) In response to agonist stimulation, CdGAP is phosphorylated by RSK on Ser1093 and Ser1163, which permits the recruitment and binding of 14-3-3 proteins. (**B**) The interaction 14-3-3/CdGAP inhibits the nucleocytoplasmic shuttling of CdGAP, leading to CdGAP sequestration in the cytoplasm, inhibition of the GAP activity and E-cadherin repression. (**C**) Consequently, Rac1-GTP levels are increased, inducing cell spreading, and E-cadherin expression is elevated.

In this report, we provide evidence for a novel mechanism of regulation of CdGAP activity and subcellular localization by RSK-dependent phosphorylation and interaction with 14-3-3. We demonstrate that CdGAP is phosphorylated at the C-terminal region on Ser1093 and Ser1163 residues by RSK, which creates docking sites for 14-3-3 binding. We show that 14-3-3 binding to these phosphosites sequesters CdGAP in the cytoplasm to inhibit the GAP activity of CdGAP towards Rac1 and its ability to repress E-cadherin expression (Figure 10). Previous studies have identified CdGAP as a molecular target of the Ras/MAPK pathway in response to serum and PDGF [20, 24, 25]. Indeed, we identified Thr776 in the proline-rich domain of CdGAP as a major phosphorylation site of ERK1/2, which negatively regulates the GAP activity of CdGAP towards Rac1 [25]. In addition, this consensus ERK1 regulatory site is phosphorylated by GSK-3β in serum-starved cellular conditions [24]. Here, we report that CdGAP is also connected to the Ras/MAPK pathway via its phosphorylation by RSK, which is a downstream ERK effector involved in the control of cell proliferation, survival, and motility [26, 29, 35]. The Ser1093 and Ser1163 residues located in the C-terminal region of CdGAP appear to be the major phosphorylation sites of RSK, although we cannot rule out the possibility that other residues may be RSK targets as well. According to phosphosite.org, phospho-Ser1163 has been reported in 25 large-scale proteomic studies, including the phosphoproteomic analysis of breast cancer, lung cancer, and the liver [36–39]. Moreover, phospho-Ser1093 was reported in two large-scale proteomic studies, including the phosphoproteome of the liver [40, 41], and more recently, this phosphosite was identified in a comparative phosphoproteomic analysis of VEGF and angiopoietin-1 signaling in endothelial cells [42]. Of interest, we have recently shown that CdGAP is a critical regulator of VEGF-mediated signaling in angiogenesis [11]. Therefore, phospho-Ser1093 and Ser1163 at the C-terminal tail of CdGAP appear to be regulated by various agonists in different cellular contexts.

We show that the RSK-dependent phospho-Ser1093 and –Ser1163 residues create docking sites for binding to 14-3-3β and σ adaptor proteins. The 14-3-3 family of proteins consists of 7 isoforms (β, γ, ε, σ, ζ, τ, η) sharing a high degree of homology amongst vertebrates [43–45]. They are crucial regulators of several intracellular signaling pathways. They form homo- and heterodimers that interact with a variety of target proteins containing the consensus motif, RSXpS/pTXP, thereby affecting their activity, subcellular localization, and protein stability [30, 31, 46–48]. Consequently, dysregulation of 14-3-3 proteins is often associated with tumorigenesis with a 14-3-3 isoform expression signature emerging in many types of cancer [45, 49]. Because of the requirement of an Arg residue at the −3 position, 14-3-3 binding sites are often regulated by Ser/Thr basophilic protein kinases of the AGC family [29]. A recent phosphoproteomic analysis of the 14-3-3 interactome in melanoma cells has indeed identified a large number of potential RSK substrates, including CdGAP [29]. Here we show that the RSK-dependent phospho-Ser1093 and –Ser1163 are binding sites of 14-3-3β, which is supported by a phosphoproteomic study reporting these phospho-residues as 14-3-3 binding sites with excellent scores [50]. However, the residual interaction between the CdGAP-S1093A/S1163A mutant and 14-3-3β suggests that other residues may be involved in the interaction. In fact, we found that the N-terminus PBR-GAP domain and the basic region (BR) of CdGAP are also able to bind to 14-3-3β. Although Ser272 in CdGAP-BR does not mediate the interaction with 14-3-3β, we cannot exclude the possibility that additional AGC family kinase-dependent consensus sites within CdGAP-BR as indicated in Table 1 could be involved in the interaction between 14-3-3β and CdGAP. However, no phospho-dependent basic motif can be identified in the PBR-GAP domain, suggesting an interaction through an unphosphorylated residue. Interestingly, a basic motif RSKKIE similar to the unphosphorylated basic RSx_1-3_E-like motif previously reported to interact with 14-3-3 [51] could mediate the interaction between the PBR-GAP domain and 14-3-3 proteins in a phosphorylation-independent manner. Altogether, the association between 14-3-3 dimers and CdGAP may be mediated in part by the N- and C-terminus of CdGAP, though both regions may differently cooperate in the context of the full-length protein.

Furthermore, our study demonstrates that the recruitment of 14-3-3 proteins to CdGAP through the phospho-Ser1093 and S1163 residues sequesters CdGAP in the cytoplasm and inhibits CdGAP–induced cell rounding and consequently, its GAP activity towards Rac1. This regulatory mechanism of CdGAP is similar to the regulation of a number of GEFs and GAPs previously identified in global 14-3-3 interaction screens [52–54], including Deleted in liver cancer 1 (DLC1), ARHGAP22, and the RhoGEF AKAP-Lbc. Notably, the RhoGAP DLC1, a tumor suppressor protein inactivating RhoA in many types of cancer [55], is phosphorylated by PKC/PKD protein kinases on Ser residues, which create binding sites for 14-3-3 proteins. This phosphorylation results in the inhibition of the RhoGAP activity and nucleocytoplasmic shuttling of DLC1 [52]. Therefore, these studies support a general role for 14-3-3 adaptor proteins in the control of small GTPase regulators, and consequently, cytoskeletal regulation and organization. We have previously shown that PI-3 kinase activation causes the recruitment of CdGAP to the plasma membrane, likely via the binding of PI-3,4,5 to the PBR preceding the GAP domain [18]. This lipid interaction is essential for CdGAP activity to induce cell rounding. Therefore, the interaction of 14-3-3 with CdGAP may impede the recruitment of CdGAP to the plasma membrane by inhibiting the interaction between PI-3,4,5 and the N-terminus PBR-GAP domain. We also report here that CdGAP localizes to the nucleus in a proportion (35%) of transfected cells and that co-expression with 14-3-3β significantly reduces CdGAP nuclear localization. In a separate study, we have uncovered a previously unknown nuclear function for CdGAP where it functions in a GAP-independent manner as a critical E-cadherin transcriptional co-repressor with Zeb2 to promote breast tumorigenesis and metastasis to the lungs [17]. Indeed, we show here that 14-3-3β abrogates the ability of CdGAP to repress E-cadherin expression and to induce cell migration. The loss of E-cadherin expression resulting in the disruption of adherens junctions correlates with increased cell migration, invasion, and cancer metastasis [17]. Thus, 14-3-3 binding to CdGAP may behave as an important negative regulator of CdGAP transcriptional activity by cytosolic sequestration, leading to the inhibition of epithelial-to-mesenchymal transition (EMT), cell motility and invasion of breast cancer cells. Targeting 14-3-3-CdGAP interactions may help to define novel therapeutic opportunities for breast cancer treatment.

The AOS-related CdGAP-Q683X and –K1087Sx4 mutants have been first identified in autosomal-dominant AOS patients and displayed an increased GAP activity towards Cdc42, highlighting the importance of Rac1/Cdc42 regulation in the developmental processes of scalp and limb formation [21]. Moreover, we have shown that the C-terminus of CdGAP was able to interact with the N-terminal PBR-GAP domain, suggesting a mechanism of regulation of CdGAP activity by the C-terminus [21]. The results presented in this study show that the AOS-related CdGAP-Q683X and –K1087Sx4 mutants lacking the C-terminal tail displayed a higher proportion of cell rounding and lost the nucleocytoplasmic regulation by 14-3-3 proteins, which correlate with the reduction of interaction between the mutants and 14-3-3β. Therefore, these novel findings strongly support a mechanism, whereby the binding of 14-3-3 proteins to the C-terminus of CdGAP is necessary to regulate the subcellular localization, GAP activity, and transcriptional activity of CdGAP (Figure 10). We propose that impaired 14-3-3 regulation of CdGAP in AOS patients may cause profound effects during early human development. In addition, we have recently reported that vascular development is impaired in CdGAP-deficient mouse embryos, associated with superficial vessel defects and subcutaneous edema, resulting in 44% perinatal lethality [11]. Altogether, these findings unveil the importance of a tight regulation of CdGAP activity in cells and tissues, which otherwise may lead to developmental disorders and cancer.

## MATERIALS AND METHODS

### DNA constructs

pEGFPC1-mCdGAP and deletion mutants, pRK5myc-hCdGAP and AOS-related mutant, pKH3-avRSK1 wild-type (WT), pcDNA-HA-14-3-3 isoforms (β, γ, σ and ζ), pEYFP-difopein, GST-14-3-3ε wild-type (WT) and GST-14-3-3ε K49E, pRK5myc-Rac1 constructs have been previously described [9, 18, 21, 24, 25, 29, 31, 33, 34, 56, 57]. pGL2Basic-pEcad (−1008/+49) was a gift from Dr Morag Park (Goodman Cancer Centre, McGill University). CdGAP point mutants were derived from the wild-type form of CdGAP in pEGFPC1 using the QuikChange^®^ Site-Directed Mutagenesis Kit (*Stratagene*), according to the manufacturer’s instructions. The following primers were used:

S272A forward-5′ GAAAGACGAGAGAACG CCCTGCCCGAGATCGTC 3′ and reverse-5′ GACGATCTCGGGCAGGGCGTTCTCTCGTCTTTC3′), S765A (forward-5′ GGCCCAAGGAATCTCGCTCCC CCTCTTACTCC 3′and reverse-5′ GGAGTAAGA GGGGGAGCGAGATTCCTTGGGCC 3′),S1093A (forward-5′ GAAACACAGGCCGTCTGCCCTCAAC CTGGACTCTG 3′ and reverse-5′ CAGAGTCCA GGTTGAGGGCAGACGGCCTGTGTTC 3′) S1163A (forward-5′ GACAGGCCGCAGGAATGCGGCTCCTG TAAGTGTG 3′ and reverse-5′ CACACTTACAGGA GCCGCATTCCTGCGGCCTGTC 3′). All plasmids were verified by sequencing. siRNAs for RSK1/2 knockdown (RSK1 siRNA: SI02223067; RSK2 siRNA: SI00065667) (Qiagen) in MDA-MB-231 cells were described previously [34].

### Antibodies

The following antibodies were used: Anti-rabbit IgG (whole molecule), anti-CdGAP (HPA036380), anti-α-Tubulin (T5168) and anti-actin (Sigma); anti-myc (clone 9E10), anti-phospho-RSK1 (Thr359/Ser363) (Millipore); rabbit and mouse anti-GFP, anti-rabbit-conjugated Alexa-488, anti-mouse-Cy3 (Molecular Probes); anti-rabbit and anti-mouse-HRP (GE Healthcare); anti-HA, anti-14-3-3β, anti-RSK1 and anti-RSK2 (Santa Cruz Biotechnology); anti-phospho-p44/42 MAPK (Erk1/2) (Thr202/Tyr204), anti-p44/42 MAPK (Erk1/2), anti-phospho-Smad2/3 (Ser465/467), anti-Smad2/3 and anti-RXRXXpS/T [Phospho-(Ser/Thr) Akt Substrate] (Cell Signaling); anti-lamin B1 (ab16048) (Abcam).

### Cell culture, transfection, and treatment

NMuMG mammary epithelial cells were grown in DMEM supplemented with 10% Fetal Bovine serum (FBS) (Wisent), 10 mM HEPES, 1 mM sodium pyruvate, 1 mM L-glutamine, 10 μg/ml insulin and antibiotics as previously described [15]. HEK293, COS-7, and MDA-MB-231 cells were cultured in DMEM supplemented with 10% FBS and antibiotics in a humidified incubator at 37°C with 5% CO_2._ HEK293 and COS-7 cells were transfected with the indicated constructs using linear polyethylenimine (PEI) (Polysciences) at a 1:8 ratio (cDNA:PEI) following the manufacturer’s instructions. For siRNA transfections, MDA-MB-231 cells were transfected with siRNAs targeting RSK1/2 or a scrambled control siRNA duplex using Lipofectamine RNAiMAX (Invitrogen) according to the manufacturer’s protocol. The final concentration of both siRNAs was 30 nM. Thirty hours post-transfection, cells were serum-starved for 18h and stimulated for 30 min with either 20% FBS, 200 nM PMA (phorbol-12-myristate-13-acetate, Cell Signaling) or 5 ng/ml recombinant Human TGF-β1 (Invitrogen). For PMA and BI-D1870 (Stemgent) treatments, cells were treated for 30min with PMA (200nM) and BI-D1870 (20nM) following a pre-treatment with BI-D1870 (20nM) for 1h.

### Immunoprecipitation, western blotting, and quantitative densitometry

NMuMG cells were lysed in lysis buffer containing 20 mM Tris-HCl, pH 7.4, 100 mM NaCl, 1mM phenylmethylsulfonyl fluoride (PMSF), 1% Triton X-100, 1 mM EDTA, 1 mM sodium orthovanadate (Na_3_VO_4_), 1 μg/ml aprotinin and leupeptin, and 50 mM sodium fluoride (NaF) (BioShop). COS-7 and HEK293 cells were lysed in lysis buffer containing 25 mM Hepes pH 7.5, 1% Nonidet P-40 (NP-40), 10 mM MgCl_2_, 100 mM NaCl, 5% glycerol, 1 mM Na_3_VO_4_, 50 mM NaF, 1 mM PMSF and 1 μg/ml aprotinin and leupeptin. MDA-MB-231 cells were lysed in a lysis buffer containing 50 mM Hepes, pH 7.5, 1% NP-40, 25 mM β-glycerophosphate, 1 mM EDTA, 5 mM EGTA, 150 mM NaCl, 25 mM NaF, 15 mM pyrophosphate, 2 mM sodium orthovanadate, 10 mM sodium molybdate, 1 μg/ml aprotinin and leupeptin, and 1 mM PMSF.

Protein lysates were centrifuged at 10,000 × g for 10 min at 4°C to remove insoluble materials. For immunoprecipitation, 2-3mg of protein lysates were incubated with the indicated antibodies for 1h followed by Protein-G-Sepharose beads (GE Healthcare). Beads were washed twice with lysis buffer and heated to 95°C in SDS sample buffer. Protein samples were resolved by SDS-PAGE, transferred to nitrocellulose membranes for Western blotting with the indicated antibodies, and visualized by enhanced chemiluminescence (ECL) (Millipore). Quantitative densitometry was assessed using Image J software [58].

### GST-protein purification and pull down assays

GST-fusion proteins (GST, GST-14-3-3ε wild-type and K49E) were produced in BL21 *E. coli* as described previously [29]. For pull-down experiments, HEK293 cells transfected or not with Myc-tagged wild-type CdGAP were lysed as described above. Cellular debris were removed by centrifugation for 10 min at 13,000 × g, and the supernatant was divided equally and incubated with 10 μg of GST, GST-14-3-3ε wild-type, or GST-14-3-3ε K49E bound to glutathione beads for 2 hours. The beads were then washed 4 times with lysis buffer prior to elution with reducing sample buffer, SDS-PAGE and immunoblotting.

### Rac1 activation assay

Transfected HEK293 cells were lysed in buffer containing 25mM Hepes pH 7.5, 1% NP-40, 10 mM MgCl_2_, 100 mM NaCl, 5% glycerol, 1mM Na_3_VO_4_, 50 mM NaF, 1mM PMSF, 1 μg/ml aprotinin and leupeptin. Protein lysates were centrifuged at 10,000 × g for 10 minutes to remove insoluble materials. Myc-tagged Rac1-GTP was pulled down by incubating 2-3mg of protein lysates for 60 min at 4°C with 30 μg of purified GST-CRIB (amino acids 73-146 of mouse PAK3 fused to GST) [59] coupled to glutathione-sepharose beads (Sigma). The beads were washed three times with the lysis buffer and then boiled in SDS sample buffer. Protein samples were resolved by SDS-PAGE and transferred to nitrocellulose for immunoblotting with anti-myc antibodies. The levels of GTP-bound Rac1 were assessed by densitometry using Image J software and normalized to total Rac1 detected in total cell lysates.

### Immunofluorescence microscopy

Immunofluorescence microscopy was performed as previously described [15, 18]. Briefly, transfected COS-7 cells grown on glass coverslips were fixed in 3.7% formaldehyde (BioShop) for 10 min before permeabilization in 0.25% Triton X-100 for 5 min. Cells were then blocked for 30 min with 0.1% BSA and incubated in blocking buffer containing the indicated primary antibodies at 4°C overnight. After washing twice with PBS, cells were incubated with Alexa Fluor 488 or Cy3-conjugated secondary antibodies (Molecular Probes) with 4’, 6’-diamidino-2-phenylindole (DAPI) for nucleus staining. Coverslips were mounted on glass slides using Prolong Gold antifade reagent (Invitrogen). Cells were examined using a laser-scanning Zeiss LSM780 confocal microscope with a Plan-Neofluar 40x/0.60 oil or a Plan-Apochromat. 63x/1.40 oil immersion objective lenses and analyzed with Zen2010 software (Carl Zeiss). Colocalization analysis and Pearson’s correlation coefficient (r) were performed using Zen2010 software, analyzing > 30 cells per condition in at least three independent experiments. For quantification of cell rounding and nuclear localization, cells were imaged with a motorized inverted Olympus microscope IX81 using a 60X U PLAN S-APO oil objective lens. Images were recorded with a CoolSnap 4K camera (Photometrics) and analyzed with MetaMorph software (Molecular Devices). At least 100 cells per condition were analyzed in at least three independent experiments.

### Luciferase assays

HEK293 cells were co-transfected with human wild-type E-cadherin-luc (−1008/+49) plasmid together with the CdGAP and/or 14-3-3β constructs. The luciferase assays were performed as described previously [17].

### Cell migration assays

Migration assays were performed as previously described [15]. 2 × 10^6^ HEK293 cells transfected with the indicated plasmids were resuspended in serum-free medium and plated in the top chamber of transwell inserts. After 48 h, cells on the bottom surface of the insert were fixed in 10% formalin and stained with a crystal violet solution. Four images were taken for each transwell insert using a Nikon Eclipse TS2 microscope (X10 magnification) objective lens. Data are representative of the average pixel count of each image that was quantitated using Image J software.

### Cell cycle analysis

Cell cycle analysis of HEK293 cells transfected with empty vector or myc-CdGAP was performed as previously reported [60]. Briefly, 1 × 10^6^ cells were harvested, washed in ice-cold PBS and fixed in 70% ethanol for 1 hour at 4°C. After PBS washes, cells were treated with RNase A for 1 hour at 37°C, stained with 10μg/ml of propidium iodide (PI) in PBS and assayed with BD FACSCanto II system. The cell cycle distribution was analyzed using FlowJo Analysis software.

### Subcellular fractionation

The subcellular fractionation assays were performed as described previously [17]. Nuclear and cytoplasmic fractions were prepared using the NE-PER nuclear and cytoplasmic extraction reagents kit (Thermo Fisher Pierce) according to the manufacturer’s protocol.

### Statistical analysis

Statistical analysis was performed using a two-sample unequal-variance Student’s *t* test. Data are presented as the mean +/− SEM and the *p* value of less than 0.05 was considered to be statistically significant. Data are representative of at least three independent experiments.

## SUPPLEMENTARY MATERIALS FIGURES



## Abbreviations

AOS: Adams-Oliver syndrome
ARHGAP: Rho GTPase-activating protein
CdGAP: Cdc42 GTPase-activating protein
DLC: Deleted in liver cancer
MEMT: Epithelial-to-mesenchymal transition
ERK: Extracellular signal-regulated kinase
FACS: Fluorescence-activated cell sorting
GDI: Guanine nucleotide dissociation inhibitor
GEF: Guanine nucleotide exchange factor
GST: Glutathione S-transferase
PDGF: Platelet-derived growth factor
RSK: p90 ribosomal S6 kinase
TGFβ: Transforming growth factor beta
