# Supplementary material for: CdGAP/ARHGAP31 is regulated by RSK phosphorylation and binding to 14-3-3β adaptor protein
Source: Oncotarget. 2018 Jan 10;9(14):11646–64. doi: 10.18632/oncotarget.24126 (PMC5837747; doi:10.18632/oncotarget.24126)
Supplement: Supplementary file 1 [file oncotarget-09-11646-s001.pdf]

www.impactjournals.com/oncotarget/ Oncotarget, Supplementary Materials

CdGAP/ARHGAP31 is regulated by RSK phosphorylation and binding to 14-3-3β adaptor protein

SUPPLEMENTARY MATERIALS

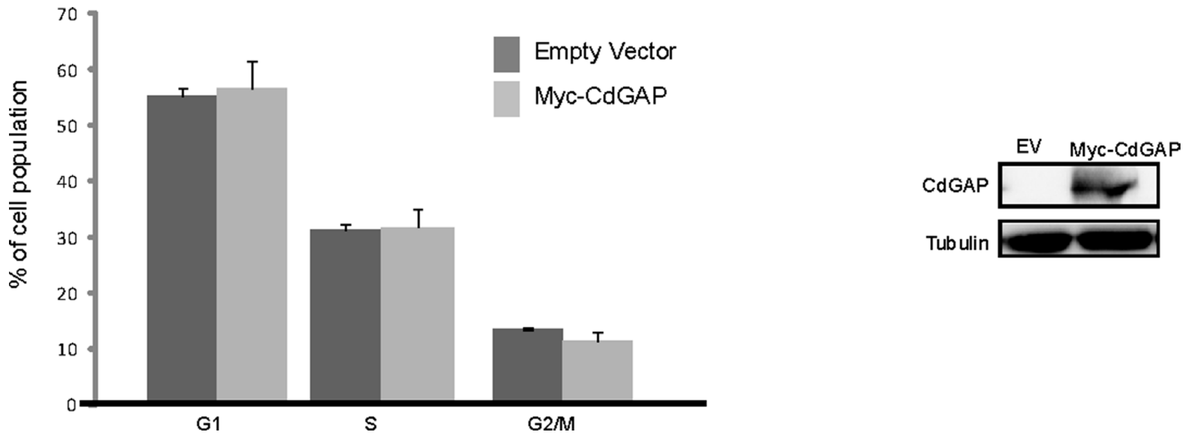

Supplementary Figure 1: Overexpression of CdGAP has no effect on the cell cycle in HEK293 cells. Cell cycle assays were performed in HEK293 cells transfected with empty vector (EV) or Myc- CdGAP. Data are presented as the mean +/- SEM. Data are representative of three independent experiments.
